# Liquid Metal Printed Zinc Tin Composite Oxide Nanosheets: A Platform for Multifunctional Sensing at Room Temperature

**DOI:** 10.1002/advs.202510017

**Published:** 2025-08-13

**Authors:** Vaishnavi Krishnamurthi, Hamidah Alluhaybi, Pargam Vashishtha, Xiangyang Guo, Huy Hoang Nguyen, Javad Khosravi Farsani, Ali Zavabeti, Sindhu Priya Giridhar, Azmira Jannat, Shimul Kanti Nath, Aaron Elbourne, Sumeet Walia, Torben Daeneke, Ylias Sabri, Chung Kim Nguyen, Nitu Syed

**Affiliations:** ^1^ School of Engineering RMIT University 124 La Trobe Street Melbourne Victoria 3001 Australia; ^2^ School of Science RMIT University 124 La Trobe Street Melbourne Victoria 3001 Australia; ^3^ Centre for Advanced Materials and Industrial Chemistry (CAMIC) School of Science RMIT University Melbourne Victoria 3001 Australia; ^4^ School of Materials Science and Engineering Southwest Jiaotong University Chengdu 610031 China; ^5^ School of Photovoltaic and Renewable Energy Engineering University of New South Wales (UNSW Sydney) Kensington New South Wales 2052 Australia; ^6^ Present address: School of Chemical and Biomolecular Engineering University of Sydney Darlington New South Wales 2008 Australia

**Keywords:** ammonia sensor, liquid metal alloy, UV optical sensor, zinc tin composite metal oxide

## Abstract

Liquid metal (LM) alloys have attracted significant interest as exceptional functional materials due to their wide range of applications. Although significant theoretical advancements have been made, the experimental investigation of surface oxides in complex metal alloys remains largely unexplored. This study investigates the formation of surface oxide in eutectic zinc (Zn)–tin (Sn) alloy to increase the understanding of composite metal oxides and enable new technological applications. The study reveals the formation of zinc tin composite metal oxide (ZTCMO) with ≈82.7 at% ZnO and ≈17.3 at% SnO_2_ by utilizing the liquid metal‐based van der Waals (vdW) printing approach. Structural characterizations confirm the formation of highly crystalline ZTCMO nanosheets with a wide bandgap of 3.3 eV. The ultrathin nanosheets demonstrate their practicality as an ultraviolet (UV) optical sensor with maximum responsivities of 3.84 A W^−1^ at 285 nm and 1.31 A W^−1^ at 365 nm, achieved at a low power density of 0.1 mW cm^−2^. Additionally, ZTCMO nanosheets exhibit excellent room temperature ammonia (NH_3_) gas sensing with high sensitivity and selectivity, detecting concentrations as low as 50 parts per million under UV light illumination. These findings highlight the potential of composite metal oxide‐based devices with the capability of multifunctional sensing.

## Graphical abstract

This work introduces a vacuum‐free liquid metal printing approach to create ultrathin zinc–tin composite oxide nanosheets. The material shows strong UV light responsiveness and high sensitivity to ammonia gas at room temperature, highlighting the potential of mixed metal oxides for advanced environmental sensors and optoelectronic device technologies.

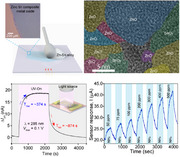

## 1 Introduction

The naturally occurring oxide layer at the interface between liquid metal (LM) and air possesses several intriguing properties, including its ultrathin nature and high surface‐to‐volume ratios, enabling many practical applications ^[^ ^1^ ^]^ particularly in supporting diverse advanced sensing activities.^[^ ^2^ ^]^ As an effective tool, liquid metal‐based printing techniques have been widely used for synthesizing few‐nanometer‐thick metal oxide nanosheets over areas of square centimeters.^[^ ^3^ ^]^ However, these methods have been predominantly applied to mono‐elemental LMs, producing oxides under various environmental conditions.^[^ ^3^, ^4^ ^]^ Expanding this frontier, the exploration of composite metal oxides could unveil new opportunities and massively expand the range of available materials. These systems exhibit surface chemistry that is either determined by the oxide with the lowest Gibbs free energy, influenced by competitive reactions and oxidation dynamics, or guided by the enthalpy of combustion, or redox processes.^[^ ^4^, ^5^ ^]^ Transitioning from a monometallic oxide to composite metal oxides often results in unique surface properties and enhanced electronic properties distinct from those found in monometallic oxides. This is highlighted in recent findings, which reveal that some bimetallic oxides often outperform their counterparts in gas sensing and nanoelectronics‐based applications.^[^ ^6^ ^]^ They also provide additional, valuable properties, such as enhanced conductivity and modulated surface chemistry, without compromising their electronic characteristics.^[^ ^6a,d^ ^]^

Among various oxide semiconductors, mixed oxides based on ZnO and SnO_2_ have emerged as highly promising materials due to their wide bandgaps and excellent functional properties.^[^ ^7^ ^]^ These oxides are widely used in a range of applications, including displays, solar cells, gas sensors, anti‐reflection coatings, and transparent conductive oxide for smart windows and photovoltaic systems.^[^ ^7a^ ^]^ Till now, mixed oxide films based on zinc and tin have been typically fabricated using techniques such as spray pyrolysis,^[^ ^7^, ^8^ ^]^ sol‐gel processing,^[^ ^7c^ ^]^ and pulsed laser deposition.^[^ ^9^ ^]^ While these methods can produce high‐quality films, they often require ultrahigh vacuum environments and suffer from slow deposition rates, which limit scalability and increase production costs.

On the contrary, LM synthesis offers a rapid, ambient‐condition route for fabricating atomically thin materials and artificial van der Waals (vdW) heterostructures, many of which are challenging or inaccessible through conventional fabrication techniques. This approach enables the formation of wafer‐scale, ultrathin oxide layers within minutes, significantly improving efficiency and scalability.^[^ ^3a^ ^]^ In contrast to the abundant work on monometallic metal oxides, however, investigations of composite metal oxide systems are surprisingly rare. In particular, the surface properties and potential applications of mixed oxides derived from Zn–Sn alloys have not been thoroughly investigated. We, therefore, explore the Zn–Sn alloy and its derived oxides as an ideal material platform. The Zn–Sn system offers several key advantages: Its eutectic alloy has a significantly lower melting point (≈198.85 °C) compared to pure Zn or Sn, allowing for more energy‐efficient synthesis.^[^ ^10^ ^]^ It also enables the formation of uniform, ultrathin mixed oxide films directly from the liquid metal surface. Additionally, lattice mismatch between SnO_2_ and ZnO, while often seen as a limitation, can actually introduce beneficial interfacial defects that enhance gas sensing performance through increased surface reactivity and charge modulation.^[^ ^11^ ^]^ This emerging class of materials could be the key to next‐generation sensors and devices, sparking a new frontier in material science.

To address this critical research gap and harness the exceptional properties of both binary SnO_2_ and ZnO, we have synthesized ultrathin, large‐area zinc‐tin composite metal oxide (ZTCMO) nanosheets. These nanosheets were derived by delaminating the surface oxide layer of a molten eutectic Zn–Sn alloy, comprising ≈82.7 ± 0.9 at% ZnO and the remaining SnO_2_. Optical sensors based on ZTCMO nanosheets exhibit strong UV absorption and a notable photoresponse at room temperature (RT). The device achieved a photoresponsivity of 3.84 A W^−1^ under 285 nm irradiation at 0.1 mW cm^−2^, with a response time on the order of seconds. Moreover, the nanosheets exhibited excellent sensitivity and selectivity for NH_3_ gas detection at RT under UV light illumination, showcasing their multifunctional capabilities. Overall, this study advances liquid metal‐based research by exploring new complex alloys, facilitating the exfoliation of mixed surface oxides with multifunctional applications.

## 2 Results and Discussion

### 2.1 Material Characterizations

**Figure 1:**
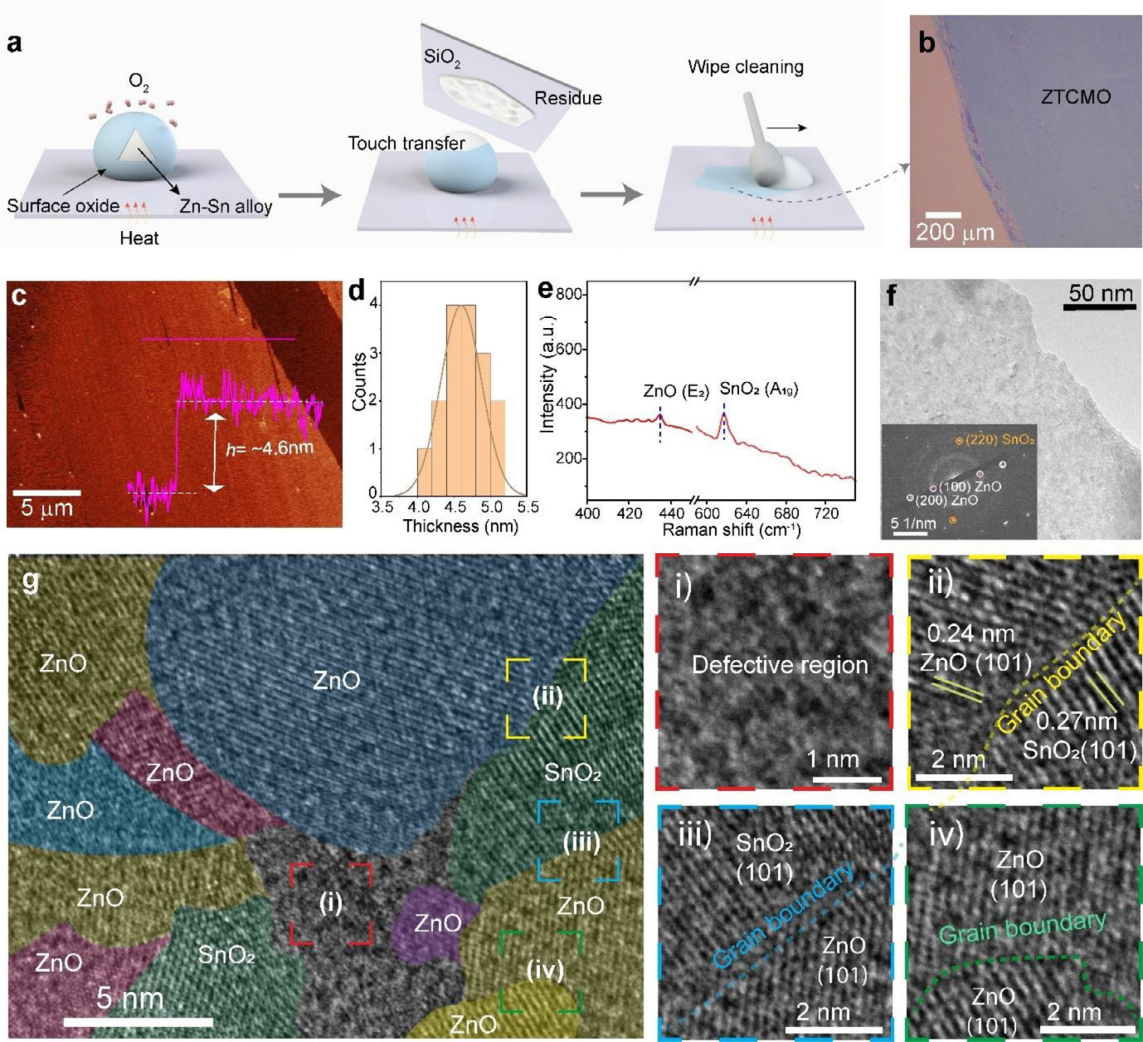
Synthesis of ultrathin ZTCMO nanosheets using the zinc–tin (Zn–Sn) liquid alloy. a) Schematic representation of the printing technique combining touch transfer and scraping process. b) Optical image of exfoliated ZTCMO nanosheets on a 300 nm thick SiO_2_/Si substrate. c) AFM image with the corresponding step height profile (inset graph) of the ZTCMO nanosheet. d) Thickness analysis at various edge locations collected from multiple nanosheets results in an average thickness of 4.75 ± 0.2 nm (standard deviation). e) Raman spectrum of ZTCMO nanosheet. f) TEM micrograph of ZTCMO nanosheet with the corresponding SAED pattern (inset bottom) of ZTCMO. g) HRTEM image with color‐coded areas illustrating different crystal grains of both ZnO and SnO_2_ within the nanosheet, (i–iv) selected zoom‐in areas indicating the presence of defective regions and grain boundaries.

Ultrathin ZTCMO nanosheets were synthesized from a molten zinc‐tin (Zn–Sn) alloy in its eutectic composition using a liquid metal‐based van der Waals (vdW) transfer method. This innovative approach combines liquid metal touch printing with the scrape printing process. Initially, the Zn–Sn alloy is melted on a hot plate at 280 °C. A thin metal layer is then deposited onto the desired substrate by gently touching it with a molten alloy droplet. Finally, the metal layer is removed using a simple scraping tool, such as a cotton swab. In this process, the weak interaction between the liquid metal alloy and its oxide layer, coupled with the strong interaction between the oxide layer and the substrate, facilitates the easy removal of the molten metal layer.^[^ ^3^, ^12^ ^]^ This leaves behind ultrathin ZTCMO nanosheets on the substrate (**Figure** **1**a). This synthesis process is performed in a vacuum‐free environment, enabling the direct transfer of the nanosheets onto desired substrates (details are provided in the Experimental Section). This unique approach consistently yields large‐area (several millimeter‐scale) ZTCMO nanosheets, as evident in the microscopic image shown in Figure 1b and Note S1 (Supporting Information).

The thickness of the synthesized ZTCMO nanosheets was determined using atomic force microscopy (AFM), which revealed a thickness of ≈4.6 nm, as shown in Figure 1c. The surface roughness was found to be 0.72 nm for the synthesized nanosheets (Note S2, Supporting Information). Thickness measurements on multiple regions confirmed the flatness of the nanosheets with minimal cracks or pinholes, supporting a well‐controlled synthesis process. Analysis of nanosheet thickness at 16 edge locations of different samples indicates that liquid metal printing results in highly reproducible nanosheets with an average thickness of 4.75 ± 0.2 nm (Figure 1d). The exfoliated ZTCMO nanosheets exhibited Raman modes for ZnO and SnO_2_, as shown in Figure 1e. While a strong Raman peak near 617 cm^−1^ (A_1g_ mode) confirms the tetragonal rutile structure of SnO_2,_^[^ ^13^ ^]^ another peak near 435 cm^−1^ (E_2_ mode) confirms the hexagonal crystal structure of ZnO.^[^ ^14^ ^]^ A multi‐printing approach was employed to increase the nanosheet thickness, thereby enabling more reliable X‐ray diffraction (XRD) analysis (Note S3, Supporting Information). The resulting XRD patterns confirmed the presence of hexagonal ZnO, as indicated by a prominent peak at 36.50° (CIF File 1011258), which aligns with the film's composition of ≈82.7 ± 0.9 at% ZnO. However, no distinct peaks corresponding to SnO_2_ were observed, likely due to its low density in the film, as supported by HRTEM and XPS analyses discussed in the sections below.

A transmission electron microscopy (TEM) image (Figure 1f) of the ZTCMO nanosheet shows an ultrathin and continuous flat morphology consistent with the AFM image. The selected area electron diffraction (SAED) images (Figure 1f, inset bottom) confirmed the presence of both crystalline ZnO and SnO_2_ phases. Figure 1g shows a color‐enhanced high‐resolution transmission electron microscopy (HRTEM) image that clearly illustrates the presence of mixed crystalline phases within the ZTCMO nanosheets. Distinct nanoscale domains of both ZnO and SnO_2_ are observed, confirming their coexistence in a single sheet. However, a higher density of ZnO nanograins relative to SnO_2_ is observed. The HRTEM analysis also highlights the presence of defect‐rich regions and well‐defined grain boundaries within both oxide phases, as shown in Figure 1g(i–iv). Furthermore, lattice fringe analysis reveals that the ZnO domains exhibit a lattice spacing of 0.24 nm, corresponding to the (101) plane of its hexagonal wurtzite structure, while the SnO_2_ domains show a spacing of 0.27 nm, matching the (101) plane of the tetragonal phase. As such, the HRTEM provides strong structural evidence supporting the formation of mixed oxide nanosheets composed of both ZnO and SnO_2_.

**Figure 2:**
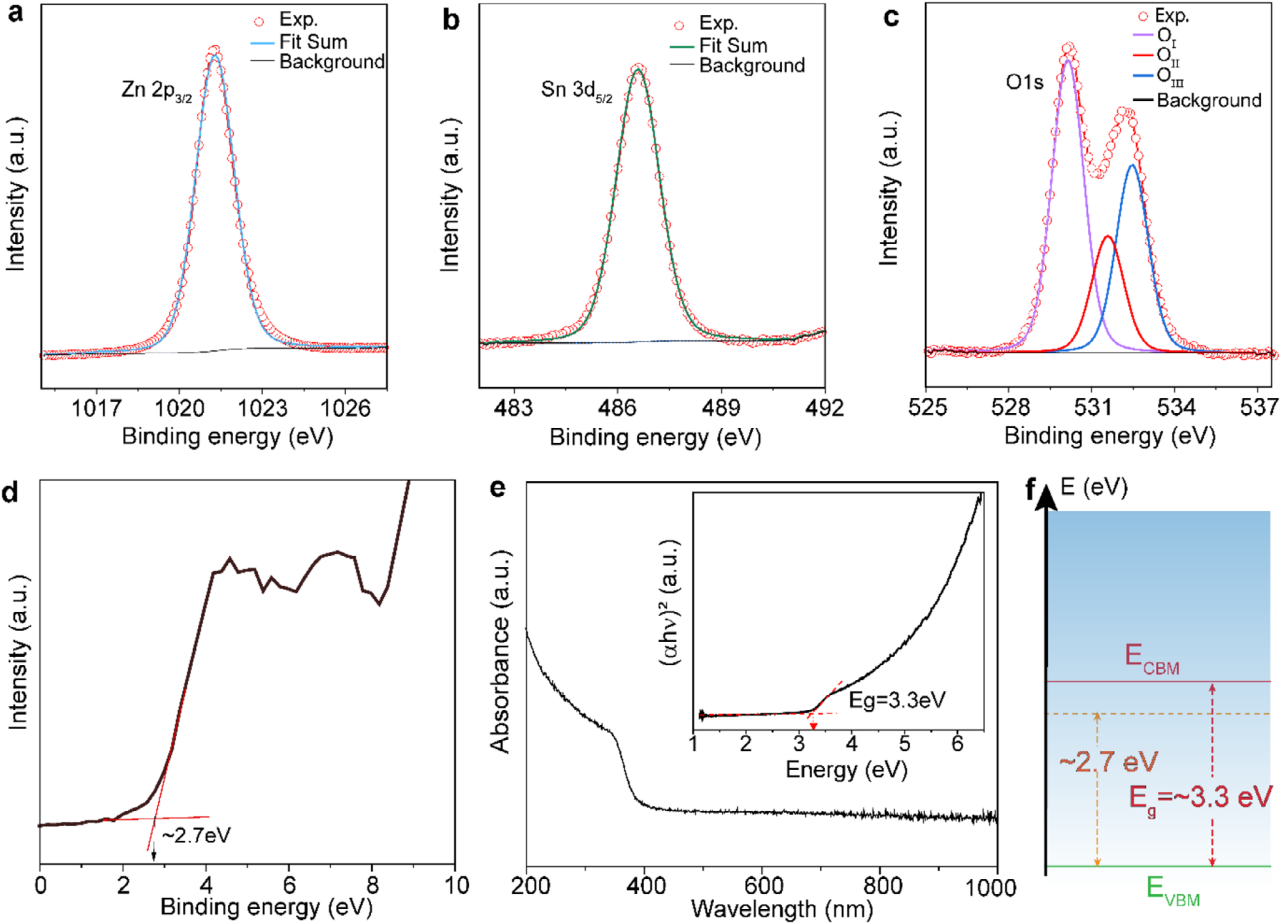
XPS elemental characterization and electronic properties of exfoliated ZTCMO nanosheets. XPS spectra of a) Zn 2p_3/2_ located at 1021.3 eV and b) Sn 3d_5/2_ located at 486.7 eV. c) O 1s confirming the presence of various oxygen species. d) XPS valence band (VB) spectra with the position of the Fermi level determined to be at 2.7 eV. e) UV–vis absorbance spectra with the cut‐off point at ≈400 nm. Tauc plot analysis (inset) illustrating the direct optical bandgaps of ≈3.3 eV. f) Simplified band diagram showing the n‐type nature of ZTCMO nanosheets.

To gain further insight into the chemical composition and the oxidation states of the synthesized ZTCMO nanosheets, X‐ray photoelectron spectroscopy (XPS) analysis was performed, and the results are summarised in **Figure** **2**a–c. Figure 2a shows a characteristic Zn 2p_3/2_ peak located at a binding energy of 1021.3 eV, featuring the Zn^2+^ states. Figure 2b displays a single component Sn 3d_5/2_ peak located at 486.7 eV, revealing the presence of only the Sn^4+^ chemical states in the exfoliated ZTCMO sample. Our XPS data confirms the absence of the lower oxidation state of tin (i.e., Sn^2+^) as identified by TEM and Raman characterizations. These results are consistent with earlier reports on ZTCMO films.^[^ ^15^ ^]^ Notably, tin oxide synthesized at 280 °C from molten tin normally comprises a mixture of SnO and SnO_2_ phases, featuring tin ions in both Sn^2^⁺ and Sn⁴⁺ oxidation states.^[^ ^13^ ^]^ In contrast, the chosen eutectic liquid metal alloy and the synthesis procedure in the present study produced only SnO_2_ phase alongside ZnO. It is noteworthy that XPS analysis did not detect any elemental Zn or Sn, highlighting the effectiveness of the cleaning procedure in eliminating residual metallic contaminants after synthesis. Figure 2c displays the deconvolved O1s spectrum, which consists of varying oxygen species. The O1s spectrum observed near 530.6 eV is assigned to the oxygen bound to either tin or zinc (O_I_), whilst the smaller peak (O_II_) near 532.2 eV belongs to oxygen near the defect sites.^[^ ^16^ ^]^ The final peak centred at 533.3 eV (O_III_) is attributed to the SiO_2_ substrate. XPS data collected from three different regions on the ZTCMO nanosheets show that the oxide layer mainly comprises 82.7 ± 0.9 at% ZnO, and the remainder consists of SnO_2_. This composition remains largely unchanged even after six months, as shown by the aged sample with 84.2 ± 1.2 at% ZnO (Note S4, Supporting Information). This ZnO‐rich surface is further confirmed by HRTEM analysis (Figure 1g), which reveals a greater presence of ZnO nanograins relative to SnO_2_.

The preferential formation of ZnO over SnO_2_ can be primarily attributed to the thermodynamic and kinetic factors influencing the oxidation process, as likely elucidated by the following reasons. Zinc has a higher oxidation tendency than tin and is more readily oxidized at elevated temperatures.^[^ ^17^ ^]^ According to Ellingham diagram predictions, zinc exhibits a more negative Gibbs free energy for oxide formation compared to tin, indicating a stronger thermodynamic driving force for ZnO formation.^[^ ^18^ ^]^ This suggests that at the synthesis temperature of 280 °C, zinc is more likely to oxidize readily and form a stable oxide layer compared to tin. Furthermore, owing to the high kinetics of Zn, a high diffusion rate is achieved, permitting Zn to diffuse rapidly along the boundaries^[^ ^19^ ^]^ of the molten alloy's surface. This fast diffusion of Zn or oxygen along Sn grain boundaries plays a dominant role in the oxidation process.^[^ ^19^ ^]^ Lastly, Zn exhibits a more negative redox potential of −0.76 V in comparison to Sn (−0.14 V), explicitly underscoring its ability to lose electrons and consequently generate a stronger driving force for oxidation and enrichment at the surface, a factor that is highly pertinent in this context as well.^[^ ^5b^ ^]^

By extrapolating the leading edge of the baseline of the valence band photoemission from the XPS valence band spectrum (Figure 2d), the difference between the valence band maximum (*E_VBM_*) and Fermi level (*E_F_*) was found to be ≈2.7 eV. The UV–vis–NIR absorbance spectrum of the ZTCMO nanosheets (Figure 2e) displays significant absorption in the UV region and high transparency across the visible region. The Tauc plot extracted from the absorption spectra resulted in a direct optical bandgap of *E_g_* ≈ 3.3 eV for the exfoliated ZTCMO nanosheets (inset of Figure 2e), revealing their wide bandgap nature. The appearance of an additional absorption feature between 350 and 400 nm could be attributed to defects due to the presence of oxygen and/or Zn^2+^/Sn^4^ ion vacancies.^[^ ^20^ ^]^ Based on the above analysis, a simplified band diagram was sketched, as shown in Figure 2f, indicating the *n*‐type semiconducting nature of the synthesized nanosheets.^[^ ^21^ ^]^ Before utilizing the synthesized composite nanosheet as a functional material for practical applications, its electrical characteristic, particularly the electron mobility, was evaluated through field effect transistor (FET) measurements (Note S5, Supporting Information). These measurements indicated a relatively low electron mobility of 1.2 cm^2^V^−1^s^−1^. This reduced mobility is primarily attributed to the grain boundaries, which act as charge carrier scattering centres, interrupting the crystal lattice continuity, and thereby limiting carrier transport.^[^ ^22^ ^]^ This interpretation is supported by the high‐resolution transmission electron microscopy, which also reveals the presence of grain boundaries and structural defects within the nanosheets (Figure 1g(i–iv)) that can impede carrier mobility.

### 2.2 UV‐Photodetection Using the ZTCMO Nanosheets

**Figure 3:**
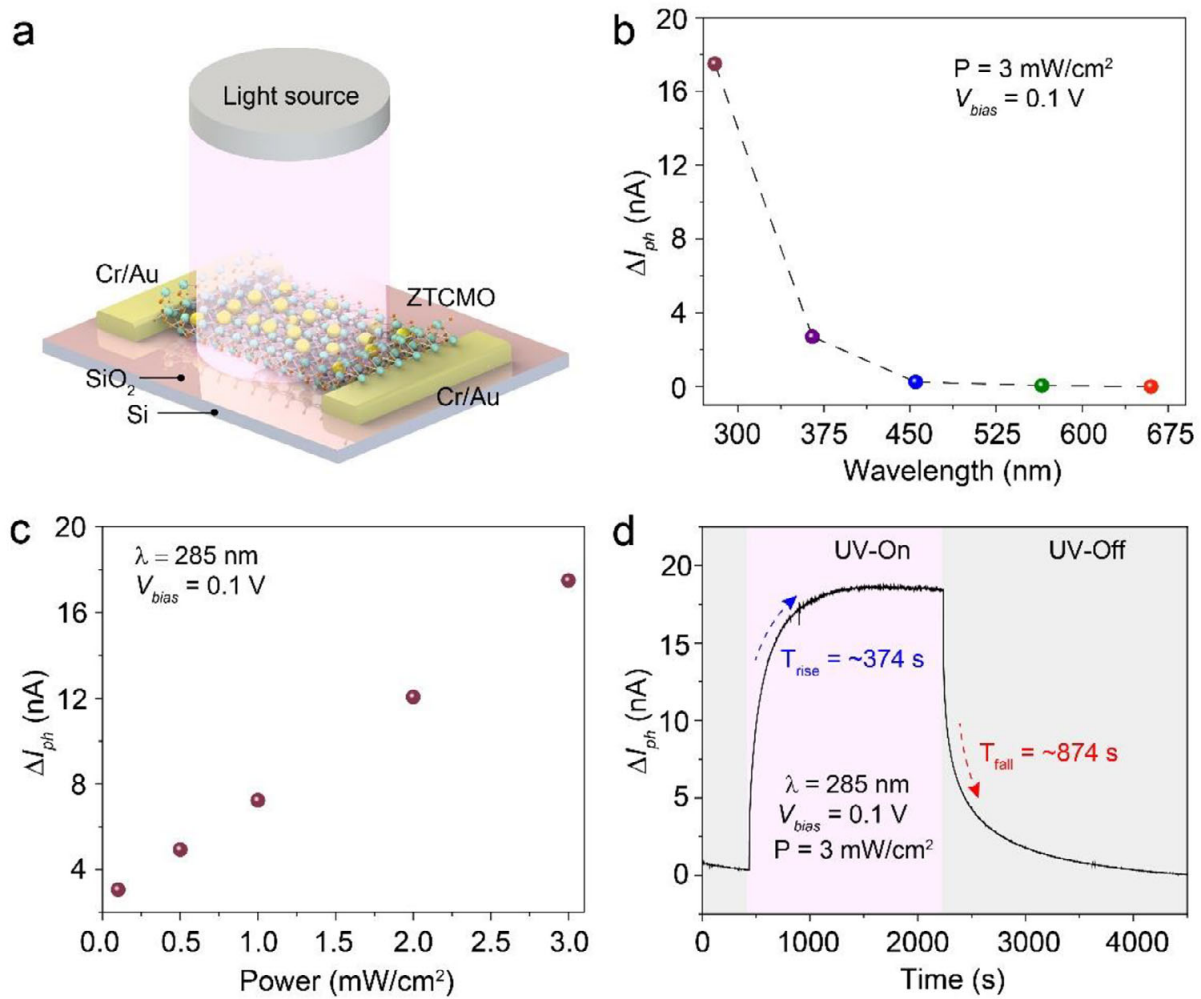
Photoresponse of ZTCMO nanosheets. a) Schematic of the optical sensor fabricated on the vdW exfoliated ZTCMO nanosheet. b) Change in photocurrent obtained at different wavelengths with a power intensity of 3 mW cm^−2^ and a bias of 0.1 V. c) Δ*I*_ph_ of different power intensities measured at 285 nm wavelength. d) Response time of the ZTCMO optical sensor under 285 nm illumination calculated at *V*_bias_ of 0.1 V and power intensity of 3 mW cm^−2^.

Taking advantage of their wide bandgap nature, the potential applications of the synthesized ZTCMO nanosheets were further investigated by fabricating a two‐terminal optical sensor. **Figure** **3**a shows a schematic of the device, with an ultraviolet (UV) light source illuminating the ZTCMO nanosheet, which acts as the active channel material. Figure 3b and Note S6 (Supporting Information) display the changes in photocurrent (Δ*I*_ph_) when illuminated with light sources ranging in wavelength from 285 to 660 nm (UV‐B to visible‐red). Furthermore, various Figures of Merit (FoM), including responsivity (R), detectivity (D*), external quantum efficiency (EQE), and response time, have been characterised. The responsivity to 285 and 365 nm illumination at a power intensity of 3 mW cm^−2^ and 0.1 V was calculated to be 0.729 and 0.112 A W^−1^, respectively, with maximum photoresponse observed at 285 nm. At the same bias and power intensity, the values of D* were obtained to be 5.21 × 10^12^ Jones for a wavelength of 285 nm and 1.41 × 10^12^ Jones for 365 nm wavelength, respectively. EQE was calculated to be 322.92% at 285 nm and a drop to 38.22 % at 365 nm. Above this wavelength, the photoresponse of ZTCMO significantly decreased, with Δ*I*_ph_ falling close to zero at relatively longer wavelengths, consistent with the large bandgap of the synthesized ZTCMO nanosheets. Additionally, the negligible photoresponse to wavelengths beyond the bandgap can be attributed to trap states in the semiconductor, which result in photogeneration and recombination events even at longer wavelengths.^[^ ^23^ ^]^ The values of *R*, D*, and *EQE* for different wavelengths are summarized in Table S1 (Note S7, Supporting Information).

As shown in Figure 3c, the photoresponse of ZTCMO nanosheets was further characterized by varying the incident light intensity, and a linear increase in *I*_ph_ was observed, attributable to the photoconductive effect.^[^ ^24^ ^]^ As evident from the figure, an increase in intensity from 0.1 to 3 mW cm^−2^ led to an increase in *I*_ph_ from 3.07 to 17.5 nA, demonstrating the exceptional photoconversion ability of the ZTCMO nanosheet obtained by the vdW synthesis technique. A maximum responsivity of 3.84 A W^−1^ at 285 nm and 1.31 A W^−1^ at 365 nm was obtained for a low power intensity of 0.1 mW cm^−2^ and a bias of 100 mV.

The obtained values for various figures of merit, therefore, highlight the excellent photoconversion capability of the devised ZTCMO optical sensor at power intensity and bias values significantly lower than those of its ZnO‐SnO_2_ counterparts with varying compositions and morphologies.^[^ ^25^ ^]^

The response speed of the device was investigated by recording the response time when illuminated with a 285 nm laser, as summarized in Figure 3d. The rise time (*T*_rise_) and fall time (*T*_fall_) were recorded, which are defined as the duration taken by the net photocurrent to increase (or decrease) from 10% to 90% (or from 90% to 10%) of the baseline value.^[^ ^26^ ^]^ In the case of the ZTCMO optical sensor, the UV‐B photoresponse curve displays a slow *T*_rise_ of ≈374 s and a *T*_fall_ of ≈874 s (Figure 3d). Previous literature reported SnO/SnO_2_ and ZnO‐based photodetectors, which exhibited relatively slow response times (Note S8, Supporting Information) similar to the synthesized ZTCMO nanosheets. The stability of an aged ZTCMO photodetector was evaluated, showing consistent and reliable photoresponse under both UV‐B and UV‐A illumination. No unusual variations were detected throughout the tests, demonstrating the device's strong cyclic and long‐term storage stability (Note S9, Supporting Information). The notably prolonged *T*_rise_ and *T*_fall_ in response to UV‐B light are attributed to defect‐induced trap states, caused by the presence of oxygen vacancies in the ZTCMO nanosheets ^[^ ^27^ ^]^ as supported by the Tauc plot analysis. Charge carriers trapped in deeper states take a considerable amount of time to be released, leading to slower response times due to the adsorption and desorption processes of photocarriers.^[^ ^28^ ^]^ Additionally, the ultrathin nature of ZTCMO nanosheets may result in increased surface defects, with the rise and fall response curves displaying persistent photoconductivity (PPC).^[^ ^29^ ^]^ Although materials with persistent photoconductivity pose challenges for fast‐response photodetectors, their long response times make them highly advantageous for neuromorphic engineering applications, including the making of artificial optoelectronic synapses, nociceptors, and olfactory systems to mimic various functionalities of the human nervous systems.^[^ ^30^ ^]^ Future studies should focus on the experimental evaluation of ZTCMO nanosheets for their potential in neuromorphic computing applications.

### 2.3 NH_3_ Sensing Using ZTCMO Nanosheets

**Figure 4:**
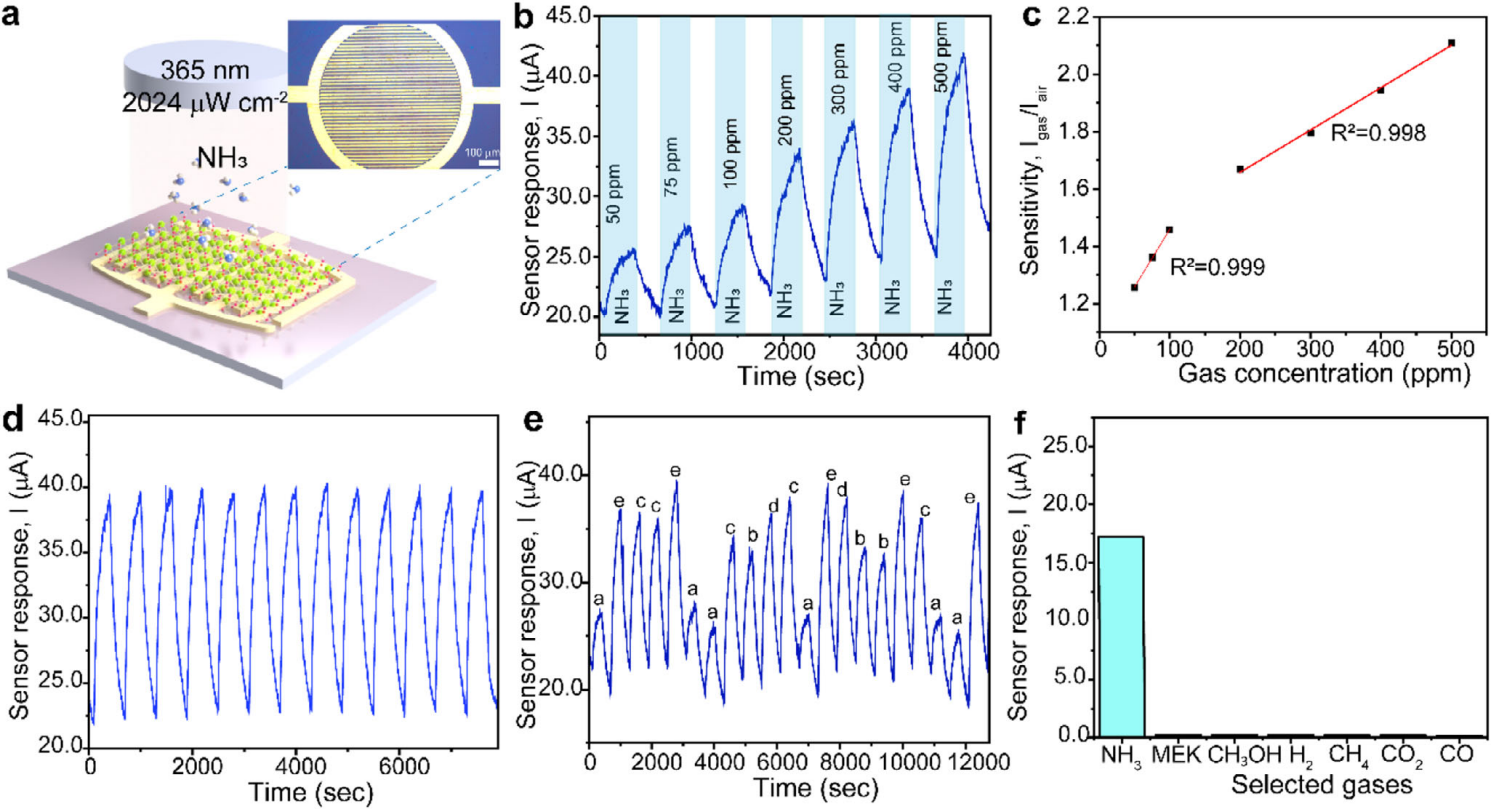
UV‐assisted RT ammonia sensing by the ZTCMO nanosheets. a) The optical image of the IDE sensor fabricated on ZTCMO nanosheets, along with the schematic structure of the sensor exposed to NH_3_ gas. b) Response profile of a UV‐assisted gas sensor towards different concentrations of NH_3_. c) Sensitivity of the sensor to different NH_3_ concentrations from 50 to 500 ppm at room temperature. d) Dynamic response–recovery cycles toward 500 ppm of NH_3_ showing stable and repeatable sensing performance. e) Response current of the sensor during the memory test. f) Sensor response displaying high selectivity towards NH_3_ and minimal cross‐sensitivity towards various other industrially relevant gas species.

Following the successful application of ZTCMO as UV optical sensors, the RT gas‐sensing performance of the nanosheets was systematically investigated. The motivation behind this approach lies in the ability of UV light to generate additional electron–hole pairs, which enhances surface reactivity and promotes more efficient gas–surface interactions.^[^ ^31^ ^]^ **Figure** **4**a illustrates the schematic diagram of the fabricated ZTCMO gas sensor device with interdigitated electrodes (see Note S10, Supporting Information, and Experimental Section for more details). The gas sensing performance of ZTCMO nanosheets was tested at RT under a 9 V bias. When the device was operated in the dark, no noticeable response was observed across different ammonia concentrations (Note S11, Supporting Information). In contrast, as can be seen in Figure 4b, the device responded well under UV illumination (365 nm wavelength and an intensity of 2024 µW cm^−2^) when exposed to NH_3_ gas with concentrations ranging from 50 to 500 parts per million (ppm). The sensor response, measured by the change in current, increased significantly under UV exposure,^[^ ^32^ ^]^ allowing it to distinguish between different concentrations of NH_3_ effectively. This improvement results from UV‐induced photocarrier generation in the ZTCMO nanosheets, which enhances the electron density and increases the active sites across the surface, making them readily available for the sensing reaction.^[^ ^33^ ^]^

The dynamic response curves of ZTCMO nanosheets at various NH_3_ concentrations showed distinguishable responses across the tested gas range (Figure 4b). The current magnitude increased from ≈5 µA at 50 ppm to ≈17 µA at 500 ppm NH_3_ concentration in the gas chamber. The observed response and recovery times for 50 ppm ammonia were approximately 145 and 166 s, respectively. While the response and recovery times for other concentrations (75–500 ppm) showed slight variations, but remained generally consistent, indicating stable sensing behavior over the tested range. The sensitivity of the sensor^[^ ^33a^ ^]^ for different NH_3_ concentrations was calculated under UV irradiation, as shown in Figure 4c. A linear calibration curve (R^2^ = 0.999) for the lower NH_3_ concentration region is observed for the ZTCMO‐based NH_3_ sensor. From the sensing performance in the low concentration region, the limit of detection (LoD) was calculated using the standard deviation of the baseline^[^ ^34^ ^]^ in the absence of the analyte (NH_3_) gas and the slope of the calibration curve. Analysis revealed LOD to be ≈5.4 parts per billion (ppb) at RT. The repeatability of the fabricated ZTCMO gas sensor was evaluated by repeatedly exposing it to multiple cycles of 500 ppm NH_3_ at RT (Figure 4d).

Figures of merit, such as the coefficient of variance (CoV) and repeatability (R%)^[^ ^32^, ^35^ ^]^ were also assessed to determine the stability of the ZTCMO gas sensor. For a 500 ppm NH_3_ concentration, the CoV of the sensor was calculated at 2.0 %, complementing a repeatability of 97.9 %, as such, highlighting the sensor's excellent reproducibility. To further explore the gas sensing performance, the memory effect^[^ ^32^ ^]^ of the sensor was investigated to determine the impact of previous sensing events on the stability of subsequent measurements by exposing the sensor to varying concentrations of NH_3_ gas. The memory test sequence involved five distinct concentrations: *a* = 50 ppm, *b* = 100 ppm, *c* = 200 ppm, *d* = 400 ppm, and *e* = 500 ppm. Each concentration, labeled as a, b, c, d, and e, was tested a minimum of two times, as shown in Figure 4e. It is seen that the magnitude of the response remained consistent across all tested NH_3_ concentrations, irrespective of the prior NH_3_ exposure levels, thereby indicating minimal memory effects in the developed sensor.

Finally, to assess the selectivity of the NH_3_ sensor, a series of tests were conducted with a range of industry‐relevant cross‐interferent gas species including carbon dioxide (CO_2_ at 10 000 ppm), methyl ethyl ketone/MEK (C_4_H_8_O at 40.1 ppm), methane (CH_4_ at 100 ppm), hydrogen (H_2_ at 500 ppm), methanol (CH_3_OH at 100 ppm), and carbon monoxide (CO at 100 ppm). The selectivity test was performed by operating the ZTCMO sensor at RT with a 9 V bias under UV irradiation. Figure 4f displays a remarkable degree of selectivity for NH_3_ at RT by displaying a significantly stronger response, much greater than other tested contaminant gases, implying minimal cross‐sensitivity. Hence, the developed RT NH_3_ sensor based on ZTCMO nanosheets eliminates the need for heating elements. This leads to a much more energy‐efficient gas sensing device, particularly suitable for low‐power applications that can be well accommodated in wearable sensors and portable devices.

A comparative summary of ammonia sensing performance for various ZnO‐ and SnO_2_‐based gas sensors reported in the literature is presented in Note S12 (Supporting Information). It is seen that most of these sensors demonstrate limited sensitivity to NH_3_ and often depend on complex fabrication techniques, engineered morphologies, or operation at high temperatures. Moreover, several of these approaches fall short in key areas such as low production cost, scalability, reproducibility, precise control over material thickness, and sensor sensitivity. It is also noteworthy that the recently published article on SnO_2_ nanosheets synthesized via the liquid metal exfoliation technique can detect NH_3_ only at elevated temperatures.^[^ ^33a^ ^]^ Unlike traditional and state‐of‐the‐art SnO_2_ sensors, the developed ZTMCO sensor achieves efficient NH_3_ detection at RT, enabled by enhanced surface reactivity and improved charge transfer characteristics. This RT sensing capability can be attributed to the incorporation of Zn ions into the nanosheets. The grain boundaries present in the ZTMCO nanosheets introduce localized defect states that act as electron‐trapping centers, thereby modifying charge transport. These boundaries also enhance gas adsorption by providing active sites for oxygen species (O_2_^−^, O^−^) and NH_3_ molecules, which further boosts the sensor's surface reactivity.^[^ ^36^ ^]^

The sensing behavior can be attributed to the effects of the interactions between NH_3_ and chemisorbed oxygen ions along electro‐ and opto‐electro‐migration in ZTCMO nanosheets.^[^ ^31^, ^36^ ^]^ In ambient air, oxygen molecules (O_2_) are adsorbed on the ZTCMO surface, extracting electrons from the conduction band to form chemisorbed oxygen species. Three types of oxygen species could form depending on the operating temperatures, such as $O_{2}^{-}$(<100 °C), *O* ^−^(100 °C to 300 °C), and *O* ^2 −^ (*>*300 °C) respectively.^[^ ^36^, ^37^ ^]^ Based on this, $O_{2}^{-}$ is the preferred oxygen species at the ZTCMO surface at RT, forming a depletion layer on the film surface, increasing the resistance. When reducing gas NH_3_ reaches the surface of ZTCMO, NH_3_ reacts with the chemisorbed oxygen species, releasing electrons back into the nanosheets, which ultimately increases the conductance of the sensor. When the device is subjected to UV illumination, additional photogenerated electron‐hole pairs are formed in the ZTCMO nanosheet.^[^ ^38^ ^]^ These photo‐generated electrons are captured by the adsorbed oxygen in the air and thus form more ionized $O_{2(h\upsilon )}^{-}$, enhancing the sensing performance by enabling increased reaction with NH_3_ molecules.^[^ ^32^, ^33^, ^38^, ^39^ ^]^

(1) $$O_{2\left(\mathrm{gas}\right)}↔O_{2\left(\mathrm{ads}\right)}$$

(2) $$O_{2\left(\mathrm{ads}\right)}+e^{-}\to O_{2\left(\mathrm{ads}\right)}^{-}$$

(4) $$O_{2\left(\mathrm{ads}\right)}+e_{h\upsilon }^{-}\to O_{2\left(\mathrm{ads}\right)}^{-}$$

(5) $$NH_{3\left(\mathrm{gas}\right)}↔NH_{3\left(\mathrm{ads}\right)}$$

(6) $$2NH_{3\left(\mathrm{ads}\right)}+3O_{2\left(\mathrm{ads}\right)}^{-}↔N_{2\left(g\right)}+3H_{2}O_{\left(g\right)}+6e^{-}$$

The enhanced sensing performance of the composite oxide is attributed to the combined effect of the formation of a depleted layer at the surface of individual oxides (SnO_2_ and ZnO) as well as the formation of a hetero‐interface between these two oxides.^[^ ^40^ ^]^ The difference in work function between the SnO_2_ and ZnO nanograins causes a unidirectional flow of electrons, ultimately aligning the Fermi levels of the two materials.^[^ ^41^ ^]^ Unlike pure metal oxides, where sensing performance is mainly influenced by the surface potential barrier, the ZTCMO sensor exhibits improved detection capabilities due to the formation of an additional space charge layer at the interface between ZnO and SnO_2_ grains.^[^ ^42^ ^]^

Moreover, grain boundaries and defect areas (as evidenced by HRTEM in Figure 1g) further contribute by offering additional adsorption sites for gas molecules.^[^ ^43^ ^]^ The mismatch in crystal lattice parameters between SnO_2_ and ZnO also leads to additional defect states or oxygen vacancies (as demonstrated in XPS in Figure 2c), leading to the enhancement of the oxygen adsorption on the surface,^[^ ^44^ ^]^ thereby significantly improving the sensing performance.^[^ ^45^ ^]^

Overall, the development of large‐area ZTCMO nanosheets enables UV‐assisted, highly selective, and sensitive NH_3_ detection at RT, preventing toxic accidents, reducing pollution, enhancing food safety, and advancing medical diagnostics.^[^ ^46^ ^]^

## 3 Conclusion

In summary, this study employed a well‐established liquid metal‐based van der Waals printing technique to fabricate ultrathin ZTCMO nanosheets from a eutectic alloy of molten tin and zinc, all within a vacuum‐free environment. The resulting ultrathin nanosheets have a composition of ≈82.7 ± 0.9 at% ZnO, and the remainder consists of SnO_2_, validating the theoretical concept that certain oxides tend to migrate preferentially to the surface in a binary liquid metal alloy. The ZTCMO nanosheets demonstrate strong potential as multifunctional sensors, exhibiting high responsiveness to UV light as optical sensors, and excellent selectivity and sensitivity toward NH_3_ gas at room temperature. These findings highlight the promise of composite metal oxide materials for functional sensing applications, enabling safer, greener, and more versatile sensor technologies. Moreover, this work lays the groundwork for future research into complex liquid metal systems with enhanced optical and sensing functionalities, including integration into stretchable and flexible platforms.

## 4 Experimental Section

### Materials

Metallic tin pellets (99.9 %) were purchased from Indium Corporation. Zn pellets (99.8 %) were purchased from Roto Metals. All solvents were purchased from Sigma‐Aldrich and used as received. 300 nm SiO_2_/Si wafers that were used to print ZTCMO nanosheets were purchased from D&X Co., Ltd. All gas cylinders for the sensing experiments were purchased from BOC.

### Preparation of Zn–Sn Alloy

Zn–Sn alloy at its eutectic concentration of 84.8 at% Sn (91 wt.% Sn) and 15.2 at% Zn (9 wt.% Zn) (Note S13, Supporting Information) was chosen to produce atomically thin ZTCMO nanosheets. The eutectic Zn–Sn alloy has a melting point of 198.85 °C. To prepare Zn–Sn alloy with the chosen eutectic composition, both precursors were mixed in a glass beaker on a hotplate setting at 500 °C for one hour in a nitrogen‐filled glovebox with oxygen, and the moisture levels were maintained at less than 0.1 ppm. As the metal precursors were melting, they were stirred intermittently using a glass rod to ensure a uniform mixture. After thorough mixing, the molten alloy was transferred to a watch glass and allowed to cool down. The solid alloy was stored in ambient air for future use.

### Synthesis of ZTCMO Nanosheets

SiO_2_/Si substrates were sonicated with acetone, isopropanol, and distilled water, each for a minute, followed by blow‐drying with compressed air. To avoid any contamination after cleaning, the substrates were left in isopropanol before proceeding with subsequent steps. The ZTCMO nanosheets were synthesized using the modified vdW touch printing technique under normal atmospheric conditions. The synthesis process involved using a laboratory hotplate heated to a temperature of 280 °C. All substrates were preheated to 280 °C by placing them on a hot plate to avoid any thermal shock. Zn–Sn alloy from the stored mixture was collected, placed on a glass slide, and then melted. Prior to touch printing, the pre‐existing oxide layer with possible air‐borne contaminants was removed by squeezing the melt with another glass slide to obtain a pristine Zn–Sn oxide surface. At this stage, the substrate was quickly brought closer to the molten Zn–Sn alloy surface, and a layer of thin metal layer was deposited onto the desired substrate. The molten metal was scraped away manually using a cotton bud dipped into the hot octanol solution to remove the Zn–Sn metal inclusions to produce the desired ZTCMO nanosheets of large lateral dimensions. As a final step, the obtained materials were rinsed several times with isopropanol and blow‐dried with compressed air.

### Material Characterization

A Leica DM2500 optical microscope was used to obtain the optical images of ZTCMO nanosheets. A Bruker Dimension Icon AFM was used to examine the morphology of ZTCMO nanosheets under the ScanAsyst‐air mode, and the Gwyddion 2.36 software was later employed to estimate the thickness of the nanosheets. A Thermo Scientific K‐alpha XPS spectrometer with a 1486.7 eV (Al Kα) X‐ray source with a concentric hemispherical electron analyzer was employed to acquire the XPS spectra. The data obtained was subsequently processed using the CasaXPS software. A Cary 60 UV–vis spectrophotometer was used to obtain UV–vis absorbance measurements for the ZTCMO samples prepared on quartz slides. A JEOL JEM‐2100F TEM equipped with a Gatan Orius SC1000 CCD camera operating at an acceleration voltage of 200 kV was utilized to obtain low‐ and high‐resolution TEM images. TEM samples were prepared by a touch‐printing method to transfer the oxide skins from molten Zn–Sn metals onto TEM grids. The TEM images were then processed and analyzed using the Gatan Digital Micrograph software package.

### Device Fabrication

Device fabrication on ZTCMO nanosheets was performed using a standard photolithography process. A Maskless Aligner – Heidelberg MLA150 was employed to pattern the source/drain electrodes with an active area of 800 µm^2^ on ZTCMO nanosheets printed on 300‐nm‐thick SiO_2_/Si substrates. The patterned source/drain pads were deposited with Cr/Au (10/100 nm) metals using the E‐beam evaporator (PVD75‐ Kurt J.Lesker). A subsequent lift‐off process was performed by immersing the samples in acetone to obtain the Cr/Au contact pads for electrical measurements. For the ammonia sensing experiments, interdigital electrodes (IDEs) with 45 pairs of electrodes (5 µm gaps) were fabricated.

### Electrical and Photodetection Characterization

A probe station operated with a Keysight B2902A Precision Source/Measure Unit was used to perform electrical measurements of the ZTCMO nanosheets. For optoelectronic measurements, commercial monochromatic light‐emitting diodes (Thorlabs, Inc.) with wavelengths of 285, 365, 455, 565, and 660 nm were used as excitation sources during the experiments. The illumination power intensities varied from 0.1 to 3 mW cm^−2^, and the bias voltage was fixed at 0.1 V. Power intensities were calibrated with a power meter (Newport Corporation). The laser beam was directed vertically onto the ZTCMO nanosheets ≈1.5 cm from a Linkam stage. The Arduino‐Uno programmable microcontroller board was used to facilitate the pulse width modulation of light sources. All optoelectronic measurements were performed in the dark under ambient conditions with the illumination of only the target wavelength.

### Gas Sensing Setup

The gas sensing experiments were performed in a custom‐made Teflon chamber, which features an optically polished quartz window lid. The fabricated sensors were mounted on a ceramic bed. For photoactivation, a UV source (365 nm LED from Edmund Optics) was used, with an incident power of 2024 µW cm^−2^. The light intensity was calibrated using a PM16‐140 power meter from Thorlabs.

The gas sensing capability of ZTCMO nanosheets was evaluated using a computer‐controlled gas delivery calibration system, specifically a multichannel mass flow controller (MFC) system. The MFC delivered gas at a controlled flow rate and maintained the required NH_3_ concentration by blending it with air through regulated on‐off cycles. This method allowed the sensor to receive pulses of varying NH_3_ concentrations.

The NH_3_ concentrations ranged from 50 to 500 ppm using a cylinder containing 0.1% NH_3_ balanced in nitrogen. The total flow rate was maintained at 200 sccm (standard cubic centimeters per minute or mL min^−1^). To establish a stable baseline, the sensor was exposed to dry air for at least 2 hrs before conducting experiments.

The sensing performance was investigated at a fixed bias voltage of 9 V with and without light illumination. The response magnitude was defined as the difference between the output current under the NH_3_ gas exposure period (*I_gas_*) and that in the air (*I_air_*): Δ*I = I_gas_ − I_air_*. The sensitivity (S) was defined as *S*  =  *I_gas_*/*I_air_*.

## Conflict of Interest

The authors declare no conflict of interest.

## Author Contributions

V.K. and H.A. contributed equally to this work.

## Supporting information



Supporting Information

## Data Availability

The data that support the findings of this study are available in the supplementary material of this article.
