# Supplementary material for: Liquid Metal Printed Zinc Tin Composite Oxide Nanosheets: A Platform for Multifunctional Sensing at Room Temperature
Source: Adv Sci (Weinh). 2025 Aug 13;12(41):e10017. doi: 10.1002/advs.202510017 (PMC12591204; doi:10.1002/advs.202510017)
Supplement: Supplementary file 1 — Supporting Information [file ADVS-12-e10017-s001.docx]

**Supplementary Information**

**Liquid Metal Printed Zinc Tin Composite Oxide Nanosheets: A Platform for Multifunctional Sensing at Room Temperature**

*Vaishnavi Krishnamurthi^1^*^§^*, Hamidah Alluhaybi^2,3^*^§^*, Pargam Vashishtha^1^, Xiangyang Guo^2^, Huy Hoang Nguyen^1^, Javad Khosravi Farsani^1^, Ali Zavabeti^1^, Sindhu Priya Giridhar^1^, Azmira Jannat^1,4^, Shimul Kanti Nath^5^, Aaron Elbourne^2^, Sumeet Walia^1^, Torben Daeneke^1^, Ylias Sabri^1,3^, Chung Kim Nguyen^1,6*^ and Nitu Syed^1*^*

*^1^ School of Engineering, RMIT University, 124 La Trobe Street, 3001 Melbourne, Victoria, Australia*

*^2^ School of Science, RMIT University, 124 La Trobe Street, 3001 Melbourne, Victoria, Australia*

*^3^ Centre for Advanced Materials and Industrial Chemistry (CAMIC), School of Science, RMIT University, Melbourne, Victoria 3001, Australia*

*^4^ School of Materials Science and Engineering, Southwest Jiaotong University, Chengdu 610031, China*

*^5^ School of Photovoltaic and Renewable Energy Engineering, University of New South Wales (UNSW Sydney), Kensington NSW 2052, Australia*

*^6^Present address: School of Chemical and Biomolecular Engineering, University of Sydney, Darlington, New South Wales 2008, Australia*

^§^ Equal contributor

*Correspondence to [nitu.syed@rmit.edu.au](mailto:nitu.syed@rmit.edu.au), [kimchung.nguyen@sydney.edu.au](mailto:kimchung.nguyen@sydney.edu.au)

**Note 1. Synthesis approach to obtain zinc tin composite metal oxide (ZTCMO) nanosheets**

**Figure S1a-d** illustrates the synthesis approach, which begins with melting a zinc-tin alloy. This molten alloy is then squeezed with a preheated glass slide and touch-printed onto a Si/SiO_2_ substrate. Finally, the metal residues are removed with a cotton swab, leaving behind the desired ZTCMO nanosheets.


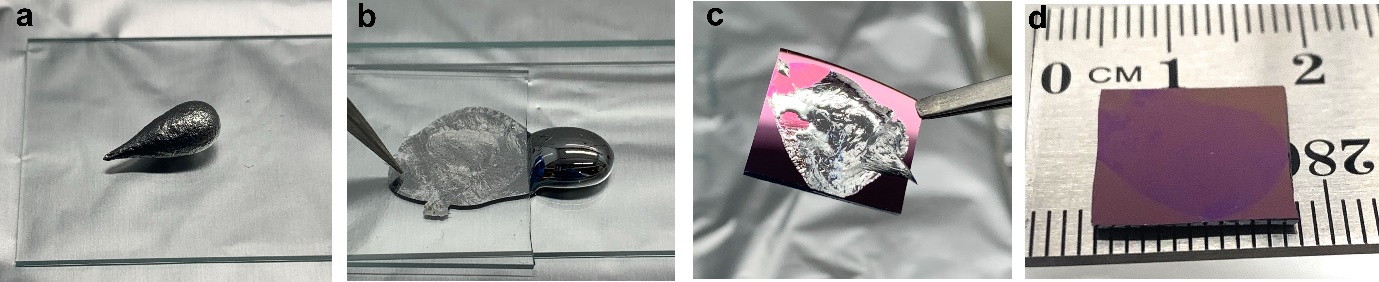


**Figure S1: Synthesis approach of ZTCMO** **nanosheets** (a) Molten zinc-tin alloy. b) Squeeze molten alloy to remove the surface contamination and pre-existing oxides. (c) Deposition of a thin layer of molten ZTCMO alloy onto the chosen substrate by touch printing. (d) A representative ZTCMO nanosheet after scraping away metal residues manually using a scraper (cotton swab).

**Note 2. AFM analysis of ZTCMO nanosheets**

The thickness uniformity of ZTCMO nanosheets was analyzed at multiple edge locations across three samples (**Figure S2a and b**). The analysis shows that the liquid metal printing produces highly reproducible nanosheets with an average thickness of 4.75 ± 0.2 nm, thereby demonstrating the thickness consistency of the nanosheets.


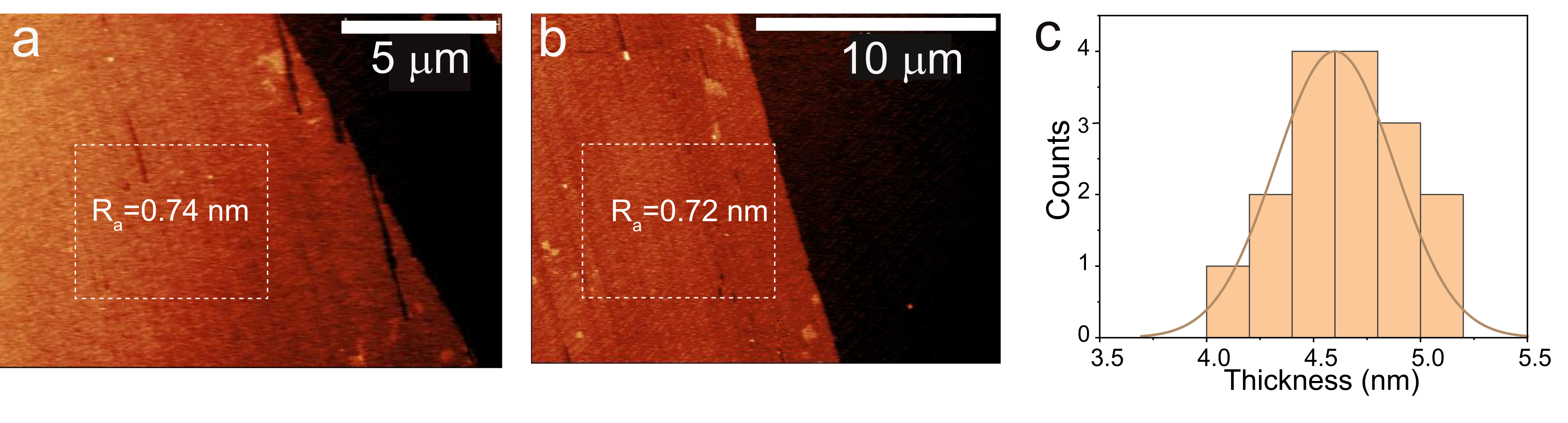


**Figure S2. Surface roughness analysis of ZTCMO nanosheets.** (a-b) AFM micrograph analysis of ZTCMO nanosheets.

**Note 3: X-ray Diffraction (XRD) analysis of ZTCMO nanosheets.**

The ultrathin nature of the as-synthesized ZTCMO nanosheets, typically just a few nanometers thick, made X-ray diffraction (XRD) analysis challenging. To facilitate the XRD characterization, a multi-printing strategy was implemented to increase the thickness of the nanosheets, converting them into thick ZTCMO films. XRD analysis revealed a prominent peak at 36.50° (**Figure S3**) that corresponds to the (101) reflection of hexagonal ZnO (CIF No 1011258). This data is consistent with the material's composition, which is approximately 82.7 ± 0.9 at% ZnO and the remaining SnO_2_. Notably, no peaks related to SnO_2_ were observed, likely due to its low concentration in the films. Alternatively, several additional peaks emerged in the XRD pattern that do not correspond to ZnO or SnO_2_ phases. Upon careful analysis, it was determined that most of these peaks are attributable to elemental Sn (CIF No 9008570, JCPDS# 004-0673#). This formation is a result of the multi-step printing process, which led to the inclusion of the metallic constituents of the Zn-Sn alloy with a composition close to the eutectic ratio - 84.8 at% Sn (91 wt% Sn) and 15.2 at% Zn (9 wt% Zn). As such, this analysis confirms the Sn-rich nature of the alloy, where a majority of the XRD reflections correspond to Sn rather than Zn-containing alloy phases.


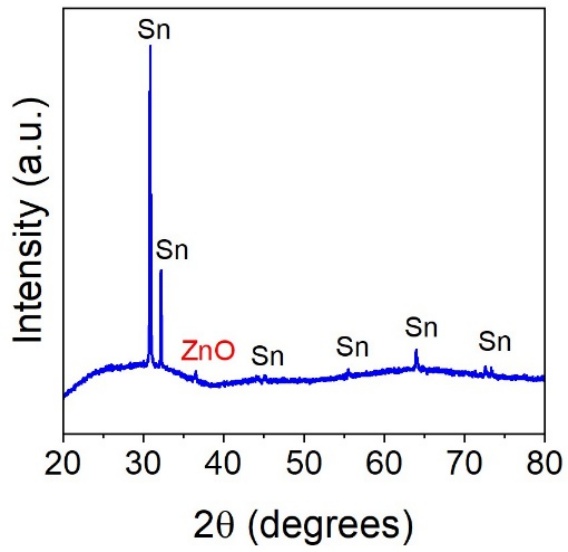


**Figure S3: XRD pattern of ZTCMO films**. XRD of ZTCMO films suggests the presence of hexagonal ZnO and elemental Sn.

**Note 4: Compositional Analysis of new and old ZTCMO nanosheets using XPS**

The composition of the synthesized ZTCMO nanosheets was confirmed through XPS analysis, conducted on three different regions on the nanosheets. The analysis revealed that the oxide layers predominantly consist of 82.7 ± 0.9 at% ZnO, and the remainder is composed of SnO_2_ (**Figure S4 a-c**). Additionally, XPS analysis was also performed on the sample that was six months old to investigate any compositional changes over time. Notably, the ZnO composition remained consistent at 84.2 ± 1.2 at% ZnO **(Figure S4d-f)**. Thereby, highlighting the compositional uniformity across different regions of the composite nanosheet as well as the compositional consistency between newly synthesized and aged samples.


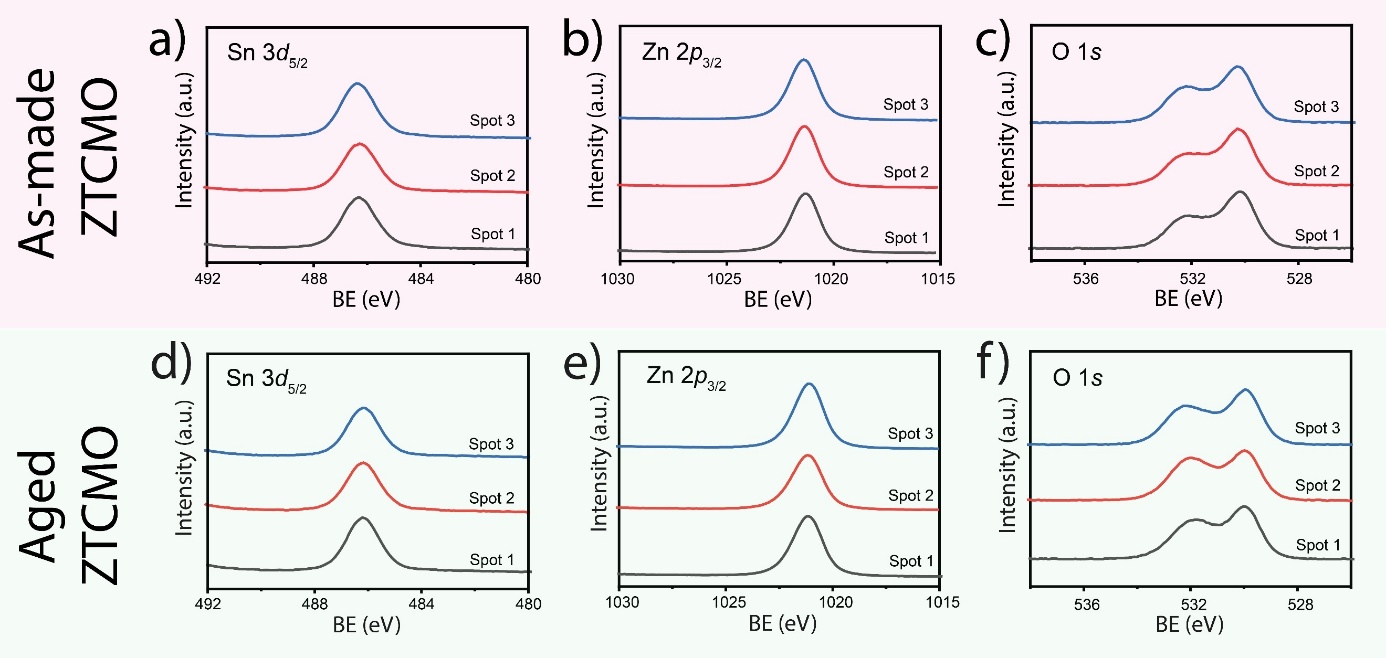


**Figure S4. XPS elemental analysis of ZTCMO nanosheets.** Measurements were conducted at three different regions of each sample. (a-c) High resolution XPS spectra of Sn 3*d*_5/2_, Zn 2*p*_3/2_, and O 1*s* for the fresh sample. The ZnO content in the fresh sample was found to be 82.7 ± 0.9 at%. (d-f) High-resolution XPS spectra of Sn 3*d*_5/2_, Zn 2*p*_3/2_, and O 1*s* for the aged sample. The ZnO content in the aged sample was found to be 84.2 ± 1.2 at%.

**Note 5: Field Effect Transistor performance of ZTCMO nanosheets**

To evaluate the electrical performance of ZTCMO nanosheets, back-gated field-effect transistor (FET) measurements were conducted on a device with ZTCMO as the active semiconducting channel. Both drain characteristics (*I*_DS_ versus *V*_DS_) and transfer characteristics (*I*_DS_ versus *V*_GS_) were measured, as shown in **Figures S5a and b**. The FET performance of the ZTCMO nanosheets demonstrates n-type behavior, with transfer curves showing an increase in drain current with an increase in the positive gate and drain voltages. The field effect mobility, estimated using Equation 1, yielded a value of 1.2 cm²/V·s.

| $\mu= \frac{L}{W}\frac{dI_{DS}}{dV_{GS}}\frac{1}{V_{DS}C_{ox}}$ | (1) |
| --- | --- |

where *V*_GS,_ *V*_DS_*,* and *I*_DS_ are the gate-source voltage, drain-source voltage, and drain-source current, respectively. The capacitance per unit between the channel and the back gate (C_ox_) was calculated to be 11.5×10^-9^ F cm^-2^ for the 300 nm thick SiO_2_ layer. The fabricated channel length (L) and width (W) were 20 and 40 µm. The reason behind this relatively low electron mobility can be attributed to grain boundaries as revealed by HRTEM.


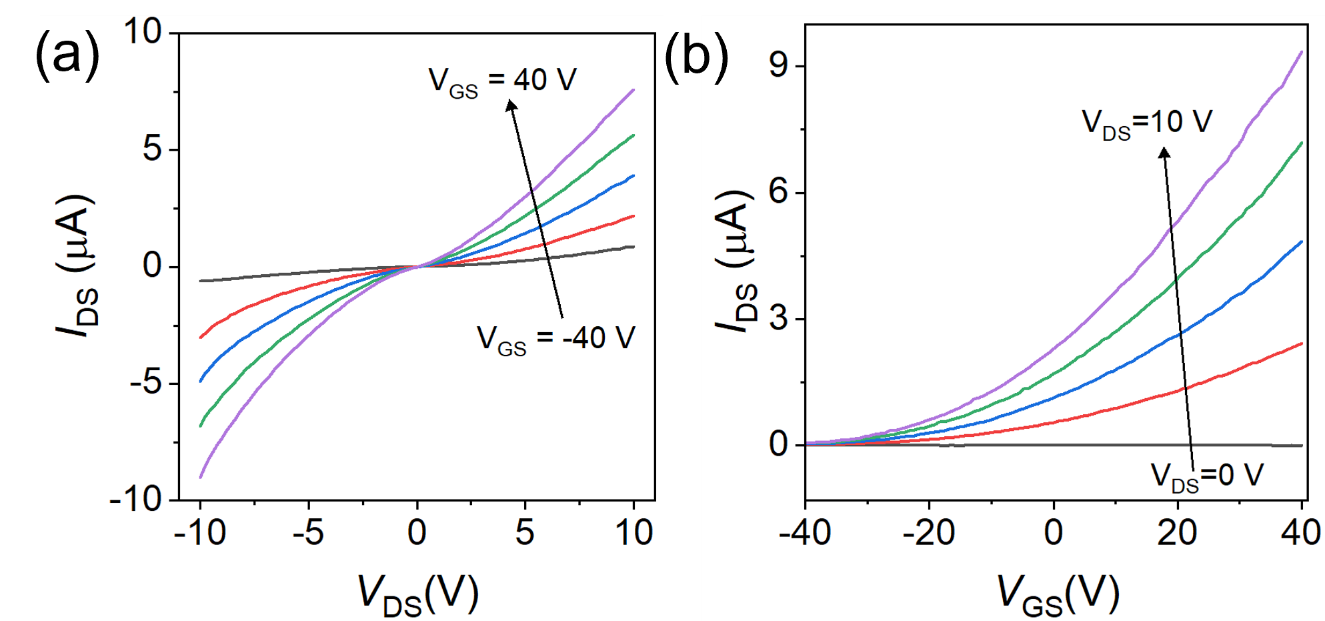
**Figure S5: Electrical characteristics of ZTCMO nanosheets.** (a) Output curves when *V*_GS_ varies from −40 to 40 V. (b) Transfer curves with *V*_DS_ ranging from 0 to 10 V.

**Note 6. Normalized transient photoresponse of ZTCMO photodetector**

**Figure S6** displays the normalized transient photoresponse of the ZTCMO device, which shows a relatively lower photocurrent under 365 nm wavelength illumination (Δ*I*_ph_ = 4.05 nA) compared to the illumination under 285 nm, which provided a Δ*I*_ph_ of 20.7 nA. At 455 nm and 565 nm illumination, Δ*I*_ph_ is further reduced, corresponding to 0.6 nA and 0.16 nA only. The device also remained unresponsive when subjected to an excitation of 660 nm.


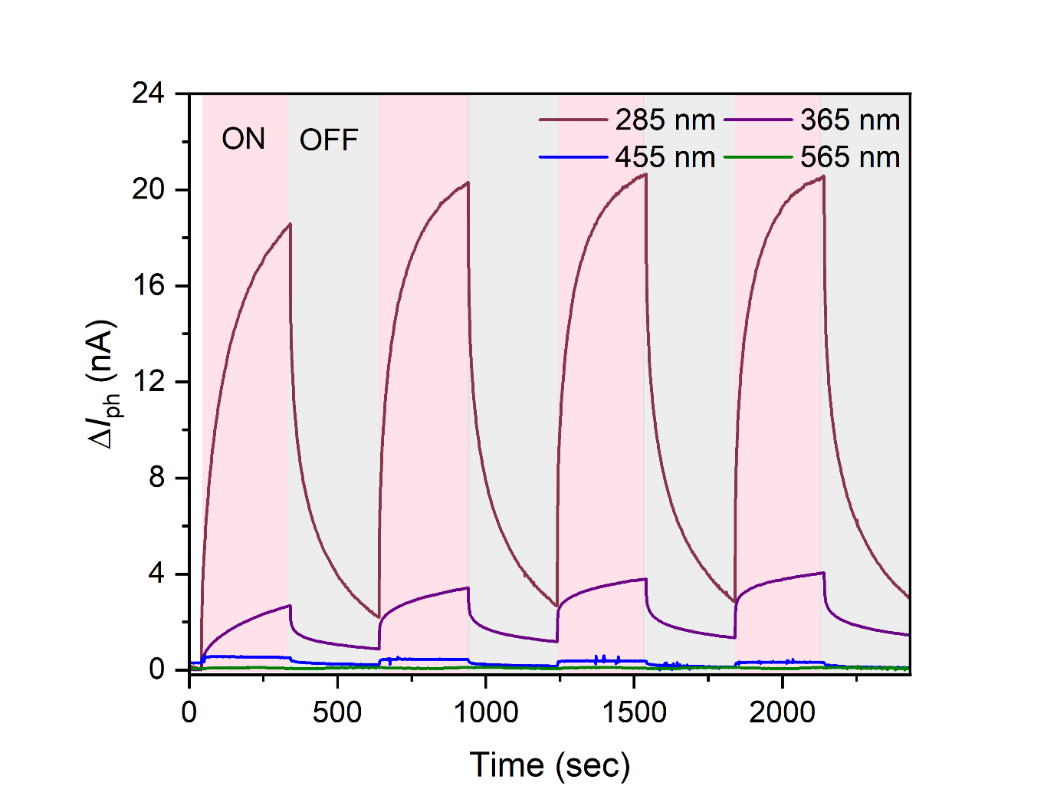


**Figure S6: Transient photoresponse of ZTCMO nanosheets.** Time resolved photoresponse of the ZTCMO photodetector when subjected to illumination under different wavelengths at a bias of 0.1 V and power intensity of 3 mW cm^-2^.

**Note 7. Responsivity, Detectivity, and External Quantum Efficiency values of ZTCMO photodetector**

Photoresponsivity (R), which determines the sensitivity of the photodetector to incident light illumination [1] was calculated to be 0.73 A W^-1^ for a 285 nm illumination at 3 mW cm^-2^ and 0.1 V incident light using equation 1.

| R$=\frac{{\Delta I}_{\mathrm{ph}}}{P_{\mathrm{inc}} A}$ | (2) |
| --- | --- |

where Δ*I*_ph_ represents the change in photocurrent, *P*_inc_ is the power intensity for a specific wavelength and A is the effective illumination area, which was 800 µm^2^ in this case.

Calculating *R* aids in determining Detectivity (*D**)[1] and External Quantum Efficiency (EQE). *D** is calculated using equation 2.

| $D^{*}=\frac{RA}{2eI_{dark}}$ | (3) |
| --- | --- |

where *R*_λ_ is the responsivity for a specific wavelength, A is the effective area of the ZTCMO nanosheets being illuminated, e is the electronic charge, and *I*_dark_ is the dark current measured from the ZTCMO device without illumination. Values of *D** were calculated to be >10^11^ Jones, revealing the high capability of the ZTCMO photodetector in detecting weak optical signals.

EQE of >10^2^ % calculated using equation 3 confirms the efficient conversion of the incident photons into free charge carriers.[1]

| $EQE =\frac{h c R}{e}$ | (4) |
| --- | --- |

where h is Plank’s constant, c is the velocity of light, R is the responsivity at a particular wavelength, e is the electronic charge, and λ is the wavelength of the incident light.

**Table S1** summarizes the values of responsivity (*R*), detectivity (*D**), and external quantum efficiency (EQE) across various wavelengths subjected to the ZTCMO photodetector at a fixed bias of 100 mV and power intensity of 3 mW cm^-2^. The figure of merit showcases their maximum value under the 285 nm illumination, demonstrating the effectiveness in photoresponse at the bandgap of the ZTCMO semiconductor.

**Table S1: R, D* and EQE values of the ZTCMO photodetector at 100 mV bias and a power intensity of 3 mW cm^-2^**

| **Wavelength (nm)** | ***R*** | ***D**** | **EQE** |
| --- | --- | --- | --- |
| **285** | 0.729 | 5.21×10^12^ | 322.92 |
| **365** | 0.112 | 1.41×10^12^ | 38.22 |
| **455** | 0.010 | 2.34×10^11^ | 2.80 |
| **565** | 0.002 | 1.27×10^11^ | 0.50 |

**Note 8. Comparison of response times with Sn and Zn-oxide based photodetectors**

Zinc oxide and tin oxide-based photodetectors developed so far have exhibited a slow photoresponse similar to that shown by the ZTCMO photodetector, as presented in **Table S2**.

**Table S2: The rise and fall times of various photodetectors show responses in the order of seconds, compared with that of the ZTCMO photodetector.**

| **Material** | **Rise time (*T*_rise_) (seconds)** | **Fall time (*T*_fall_) (seconds)** | **Ref** |
| --- | --- | --- | --- |
| ZnO nanosheets | 11.58 | 93.66 | ^[2]^ |
| SnO_2_ thin film | 55 | 102 | ^[3]^ |
| **ZTCMO nanosheets** | **374** | **874** | **(This work)** |

**Note 9: Cyclic and storage stability of the ZTCMO photodetector**

To investigate the stability of our ZTCMO photodetector device, both storage (shelf-life) and cyclic (long-term) photoresponse measurements were conducted (**Figure S7a and b**). After exposing a one-year-old device to five cycles lasting up to 40,000 seconds, the device continued to exhibit a reproducible photoresponse to periodic irregular lights of UV-B and UV-A at a bias of 0.1 V and a power intensity of 3 mW cm^-2^. The differences in photocurrent between cycles are minimal, with slight increases and decreases, indicating the one-year-old fabricated device has decent cyclic stability. No unusual changes in photoresponse were observed in the one-year-old device - with UV-B showcasing a maximum response compared to UV-A, with an expected, usual slow rise and fall time. Thereby, demonstrating the applicability of our ZTCMO photodetector for practical usage.


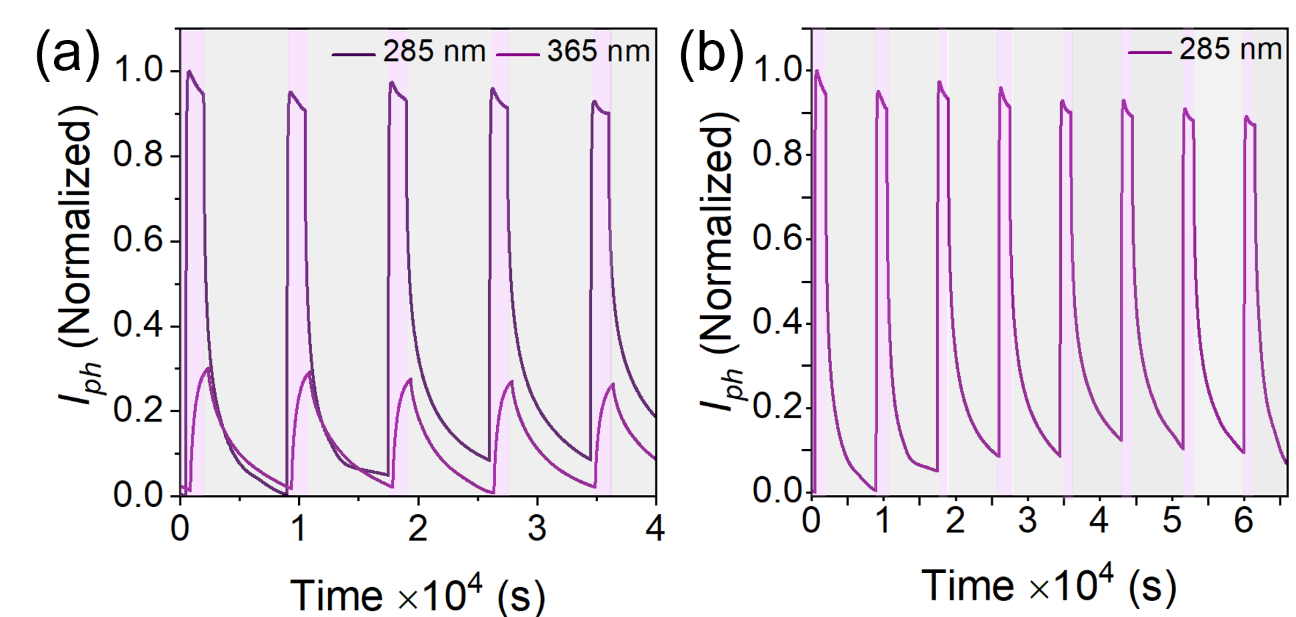


**Figure S7: Stability of ZTCMO photodetector:** (a) Shelf stability on a one-year-old device showcasing reproducible photoresponse measurements for both 285 nm and 365 nm wavelengths. (b) Long-term cyclic stability of a device stored for over six months, tested under 285 nm UV irradiation. The ZTCMO photodetector showed consistent photoresponse for more than 7 cycles. LEDs of 285 nm and 365 nm were ON for 1500 s and OFF for 7000 s.

**Note 10. Fabrication of ZTCMO devices for gas sensing**

Interdigital electrodes (IDEs) with 45 pairs of electrodes, having a gap of 5 µm, were fabricated on ZTCMO nanosheets that have large lateral dimensions, as shown in **Figures S8a and b**.


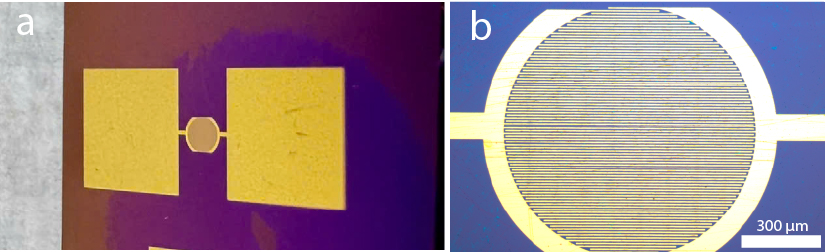


**Figure S8: IDEs on ZTCMO nanosheets** (a) Fabrication of IDEs on ZTCMO nanosheet (b) Optical image of the ZTCMO gas sensor.

**Note 11. NH_3_ detection in the absence of UV light**

When ZTCMO nanosheets were exposed to different concentrations of NH_3_ gas, the device did not show any obvious response to any of the gas concentrations in the absence of UV light, as shown in **Figure S9**, highlighting the need to use UV to assist ammonia detection.


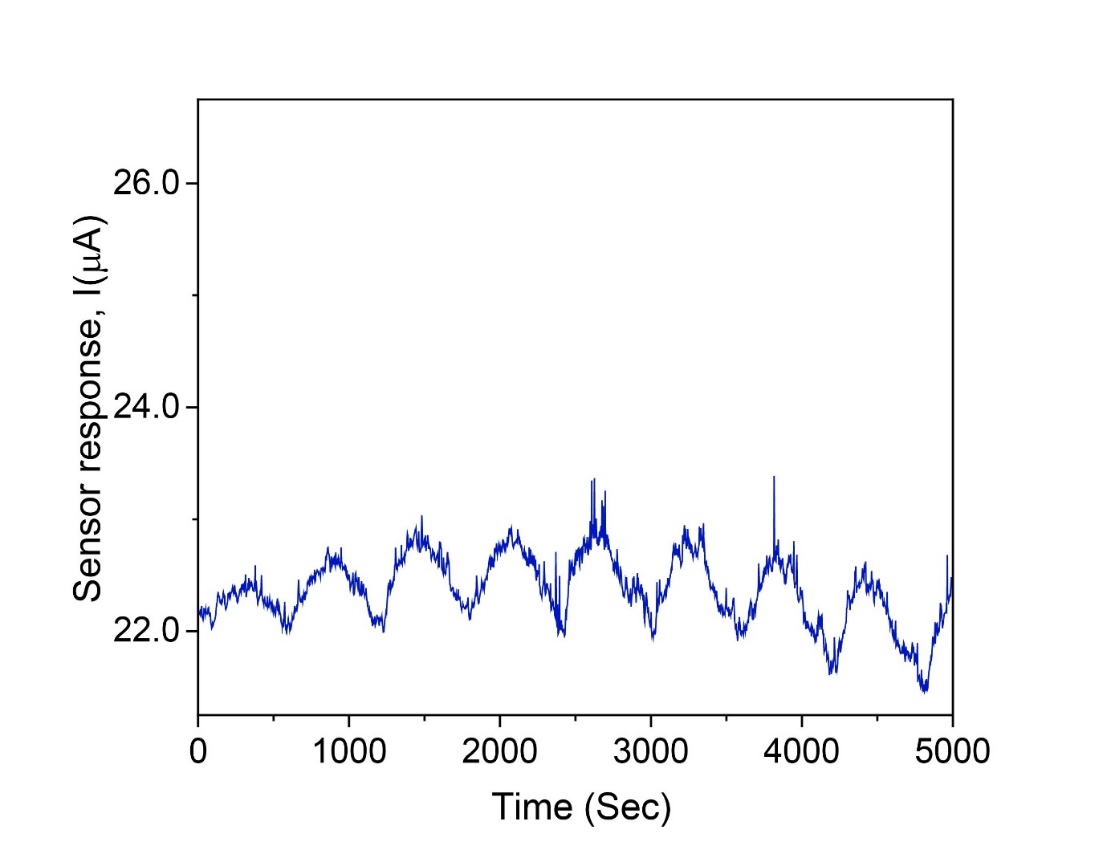


**Figure S9:** Room temperature NH_3_ sensing in the absence of UV light (NH_3_ concentrations: 50-500 ppm).

**Note 12. Comparison of the ZTCMO-NH_3_ gas sensor with other NH_3_ sensors**

**Table S3** presents a comparison of various gas sensors formulated to date with the newly developed ZTCMO NH_3_ gas sensor. The UV-assisted ZTCMO gas sensor demonstrates significantly improved response rates compared to NH_3_ sensors reported in the literature, with the benefit of operating at room temperature and utilizing ZTCMO as the sole active material for detecting NH_3_ gas.

**Table S3: Comparison of sensing parameters for NH_3_ gas detection.**

| **Material** | **Morphology** | **NH_3_ detection range (ppm)** | **Light assisted/ wavelength** | **Operating temperature (°C)** | **Sensitivity** | **LOD** | **Ref** |
| --- | --- | --- | --- | --- | --- | --- | --- |
| SnO_2_ | Nanosheet/Liquid metal printing | 5-500 | Y (UV-A) | 150 | 7.3 (at 5 ppm) | 9.43 ppb | [4] |
| SnO_2_ | Nanowire-like networks/Screen printing combined with micro-injecting and calcination (SPMIC) > 6 hours | 10-100 | No | 300 | ~1.5 (at 100 ppm) | ~~-~~ | [5] |
| SnO_2_ | Films/sol-gel | 1-800 | No | 300 | 222 (at 800 ppm) | - | [6] |
| ZnO | Nanoflakes/precipitation method | 0.6- 3 | No | 250 | - | - | [7] |
| ZnO | Nanorod/hydrothermal growth | 20-100 | No | 650 | 22.6 (at 100 ppm) | - | [8] |
| ZnO | Spherical structures/Spray pyrolysis | 1-500 | No | 350 | - | - | [9] |
| Sn doped ZnO | Fibers/thermal evaporation process | 10-200 | No | 250 | 0.0044 | **-** | [10] |
| SnO_2_: Zn films | Films/spray | 50-250 | No | RT | 121% | **-** | [11] |
| 70SnO_2_:30ZnO | Multilayer sensors for 70SnO_2_:30ZnO | 30-70 | No | RT | 0.67 (at 70 ppm) | **-** | [12] |
| **ZTCMO** | **Nanosheets** | **50 – 500** | **Y (UV-A)** | **RT** | **1.25 (at 50 ppm)** | **5.4 ppb** | **This work** |

**Note 13. Phase diagram of Zn-Sn alloy**

**Figure S10** illustrates the phase diagram of the Zn-Sn alloy, which has its eutectic point at 84.8 at% Sn and 15.2 at% Zn, with a melting point of 198.85 °C.


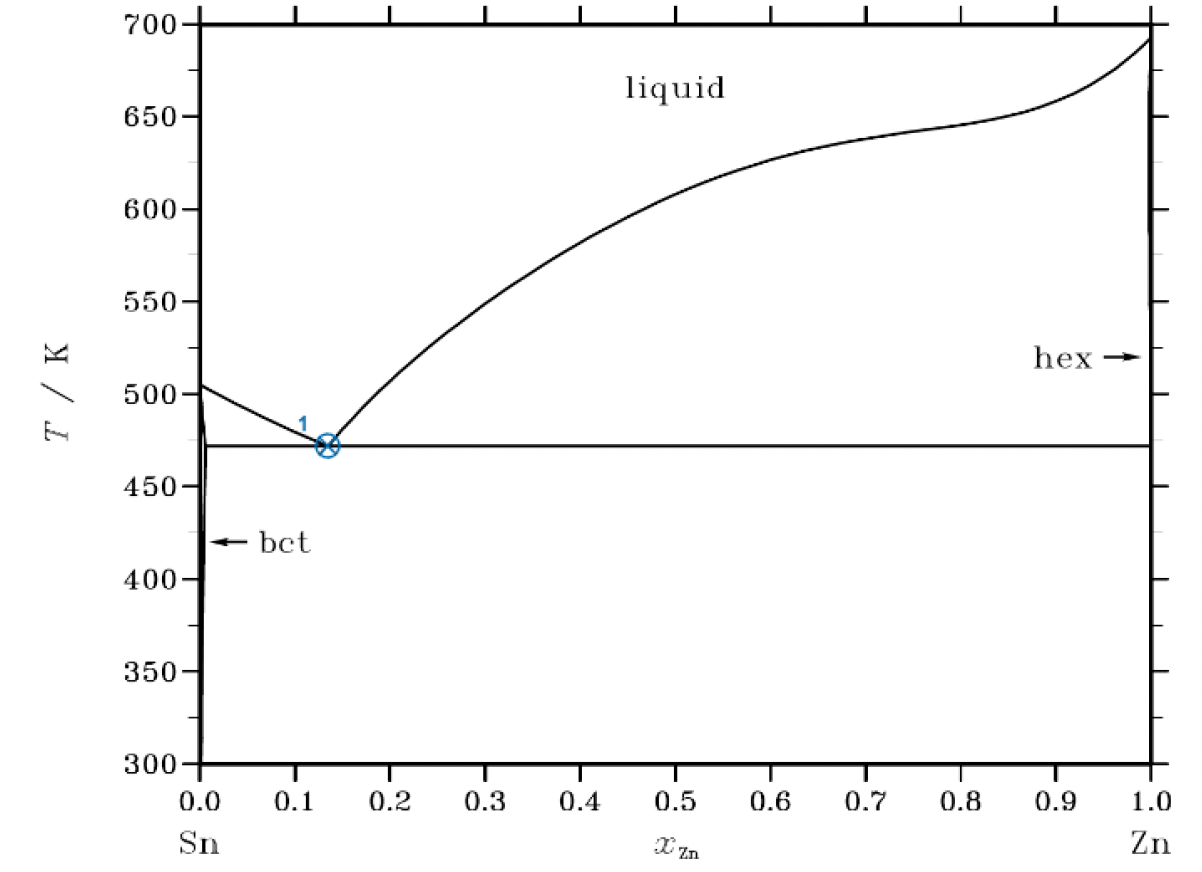


**Figure S10**: **Phase diagram of Zn-Sn alloy**. Adapted from Springer Nature.[13]

# References

[1] M. Long, P. Wang, H. Fang, W. Hu, *Adv. Funct. Mater.* **2019**, *29* (19), 1803807.

[2] V. Krishnamurthi, T. Ahmed, M. Mohiuddin, A. Zavabeti, N. Pillai, C. F. McConville, N. Mahmood, S. Walia, *Adv. Opt. Mater.* **2021**, *9* (16), 2100449.

[3] R. Djamil, K. Aicha, A. Souifi, D. Fayçal, *Thin Solid Films* **2017**, *623*, 1.

[4] C. K. Nguyen, P. D. Taylor, A. Zavabeti, H. Alluhaybi, S. Almalki, X. Guo, M. Irfan, M. A. Kobaisi, S. J. Ippolito, M. J. Spencer, S. Balendhran, A. Roberts, T. Daeneke, K. B. Crozier, Y. Sabri, N. Syed, *Adv. Funct. Mater.* **2024**, *34* (31), 2309342.

[5] S. Yi, S. Tian, D. Zeng, K. Xu, X. Peng, H. Wang, S. Zhang, C. Xie, *Sensors Actuators B: Chem.* **2014**, *204*, 351.

[6] C. S. Rout, M. Hegde, A. Govindaraj, C. Rao, *Nanotechnology* **2007**, *18* (20), 205504.

[7] S. Kanaparthi, S. G. Singh, *Materials Science for Energy Technologies* **2020**, *3*, 91.

[8] S. Anantachaisilp, S. M. Smith, C. Ton-That, T. Osotchan, A. R. Moon, M. R. Phillips, *J. Phys. Chem. C.* **2014**, *118* (46), 27150.

[9] K. Venkatesh, K. S. Varshini, B. R. Frances, B. Jeyaprakash, D. Balamurugan, *J. Electron. Mater.* **2025**, 1.

[10] S. K. Sinha, *Sensors Actuators B: Chem.* **2015**, *219*, 192.

[11] M. Boomashri, P. Perumal, A. Khan, A. M. El-Toni, A. A. Ansari, R. K. Gupta, P. Murahari, K. D. A. Kumar, *Surfaces and Interfaces* **2021**, *25*, 101195.

[12] G. Lamdhade, K. Raulkar, S. Yawale, S. Yawale, *Indian Journal of Physics* **2015**, *89*, 1025.

[13] Sn-Zn Binary Phase Diagram 0-100 at.% Zn: Datasheet from "PAULING FILE Multinaries Edition – 2022" in SpringerMaterials (<https://materials.springer.com/isp/phase-diagram/docs/c_0902137>). P. Villars, H. Okamoto, Eds.: Springer-Verlag Berlin Heidelberg & Material Phases Data System (MPDS), Switzerland & National Institute for Materials Science (NIMS), Japan.
